# Effect of Different Aggregates on the Mechanical Damage Suffered by Geotextiles

**DOI:** 10.3390/ma12244229

**Published:** 2019-12-17

**Authors:** David Miranda Carlos, José Ricardo Carneiro, Maria de Lurdes Lopes

**Affiliations:** Construct-Geo, Faculty of Engineering, University of Porto, Rua Dr. Roberto Frias, 4200-465 Porto, Portugallcosta@fe.up.pt (M.d.L.L.)

**Keywords:** geosynthetics, geotextiles, mechanical damage under repeated loading, degradation, mechanical behavior, hydraulic behavior

## Abstract

The installation process of geosynthetics can be, in some applications, one of the most relevant degradation mechanisms of these construction materials, affecting their performance and useful lifetime. In this work, three nonwoven geotextiles with different masses per unit area were submitted to mechanical damage under repeated loading tests with *corundum* and with different natural aggregates. The damage occurred in the geotextiles was evaluated by visual inspection and by monitoring changes in their short-term tensile and puncture behaviors (mechanical properties) and in their water permeability behavior normal to the plane (hydraulic property). The mechanical damage under repeated loading tests provoked relevant changes in the mechanical and hydraulic properties of the geotextiles. These changes depended on the mass per unit area of the geotextiles and on the characteristics of the aggregates. The results enabled the establishment of a correlation between the loss of mechanical strength and the variation of the water permeability normal to the plane of the geotextiles.

## 1. Introduction

Geosynthetics are polymeric materials widely used in the construction of many geotechnical, geoenvironmental, hydraulic, and transportation engineering structures. These materials are rapid and easy to install, have a relatively low cost (when compared to traditional construction materials), can avoid the use of natural construction materials (soils, aggregates, etc.) and allow the construction of structures with low visual impact on the landscape [1]. The geosynthetics are available in a wide range of forms (e.g., geotextiles, geomembranes, geogrids, or geocomposites) and can perform many different functions, like: drainage, filtration, protection, separation, reinforcement, fluid containment or erosion control.

One of the main questions about the application of geosynthetics in civil and environmental engineering works is their durability. The expected lifetime of these materials can vary from one to over a hundred years and throughout that period the geosynthetics have to perform the functions for which they were installed. In order to ensure that the materials will function for the required period of time, it is necessary to predict which conditions they will be exposed to and how those conditions will affect their properties over time. For this purpose, degradation tests of geosynthetics under accelerated conditions or under real conditions are often performed [2]. The most common degradation agents of geosynthetics include: chemical substances like acids or alkalis, oxygen, high temperatures ultraviolet radiation and other weathering agents, creep, or abrasion. The installation process can also induce some damage to the geosynthetics [2].

The installation process can be, in some applications, responsible for relevant changes in the properties of the geosynthetics. The damage that occurs during installation is mainly originated by handling the geosynthetics and by the placement, spreading and compaction of filling materials over them [1,3]. There are cases in which the materials can even be submitted to higher stresses during installation than during service life [4]. The installation procedures can provoke cuts in components (e.g., filaments or fibers), holes, tears, punctures, abrasion and, in the worst-case scenario, the total destruction of the geosynthetics [3]. The damage occurred in the installation process depends on many factors, such as: the characteristics of the geosynthetics, the grain size distribution of the soils or aggregates, the angularity and thickness of the filling materials, the compaction energy, and the use, or not, of adequate installation procedures [5,6,7,8].

The installation damage of geosynthetics can be evaluated by field tests (installation under real conditions) or by laboratory tests (that try to reproduce installation damage) [2]. The laboratory damage tests (such as the method described in EN ISO 10722 [9] for inducing mechanical damage on geosynthetics) are not always able to reproduce the field installation conditions or the installation damage suffered by geosynthetics. Therefore, this work uses the term mechanical damage instead of installation damage. The degradation suffered by the geosynthetics during the field or laboratory damage tests is often evaluated by monitoring changes in their physical, mechanical (e.g., [10,11,12,13,14]), hydraulic (e.g., [15,16,17]), or long-term (e.g., [18,19]) properties. The effect of the installation process on the interface properties between geosynthetics and the contacting soils or aggregates has also been evaluated (e.g., [20,21]).

This work gives particular attention to the effect of mechanical damage under repeated loading on the short-term mechanical and hydraulic behavior of geosynthetics. Three nonwoven geotextiles with different masses per unit area were submitted to mechanical damage under repeated loading tests (hereinafter MD tests) with *corundum* (synthetic aggregate used in the method described in EN ISO 10722 [9]) and with different natural aggregates. Nonwoven geotextiles are often applied in civil engineering structures to perform functions like protection (e.g., protection of geomembranes in waste landfills or in water reservoirs), separation (e.g., separation of layers in road pavements or in railways) or filtration of fluids (e.g., filters behind retaining walls, beneath erosion control structures or around drains). Taking into account the previous functions of nonwoven geotextiles, the damage imposed by the MD tests was evaluated qualitatively by visual inspection and, quantitatively, by monitoring changes on their tensile and puncture behaviors (mechanical properties) and on their water permeability normal to the plane behavior (hydraulic property). Mechanical properties are relevant for the survivability (resistance to degradation) of the geotextiles to the installation process and, for example, for their capability to perform functions such as protection or separation. Water permeability normal to the plane has an impact on their ability to perform filtration or separation functions. 

The main goals of this work included: (1) evaluate the effect of the MD tests, not only on the tensile and puncture properties of the geotextiles but also on their water permeability normal to the plane (rarely considered for damage evaluation), (2) correlate the changes induced by the MD tests on the mechanical and hydraulic properties of the geotextiles, (3) assess the effect of mass per unit area on the survivability of the geotextiles, (4) evaluate the effect of using different aggregates on the MD tests and (5) compare the damage caused by *corundum* with the damage induced by the natural aggregates.

## 2. Experimental Description

### 2.1. Geotextiles

This work studied three nonwoven needle-punched geotextiles (designated as G120, G300, and G480) made from polypropylene fibers (the numbers included in the designations of the geotextiles correspond to the masses per unit area defined by the manufacturers). The main physical properties of the geotextiles are presented in Table 1 (the mechanical and hydraulic properties of the geotextiles can be found in the Results and Discussion section). 

The sampling and preparation of test specimens (for the characterization and degradation tests) were carried out according to the guidelines of EN ISO 9862 [22]. The specimens were collected from positions evenly distributed over the full width and length of the geotextiles (supplied in rolls), but not closer than 100 mm to the edges.

**Table 1:** Main physical properties of the geotextiles (undamaged samples).

Geotextile	Mass per Unit Area (g·m^−2^) ^1^	Thickness (mm) ^2^
G120	120 (±9)	0.79 (±0.04)
G300	325 (±11)	3.83 (±0.09)
G480	482 (±18)	3.98 (±0.14)

^1^ determined according to EN ISO 9864 [23], ^2^ determined according to EN ISO 9863-1 [24]. (95% confidence intervals in brackets).

### 2.2. Mechanical Damage under Repeated Loading Tests

The EN ISO 10722 [9] describes a method for inducing mechanical damage to geosynthetics, provoked by a granular material, under repeated loading. This standard defines the specifications of the test equipment, the test procedures, and the characteristics of the aggregate (*corundum*).

**Figure 1:**
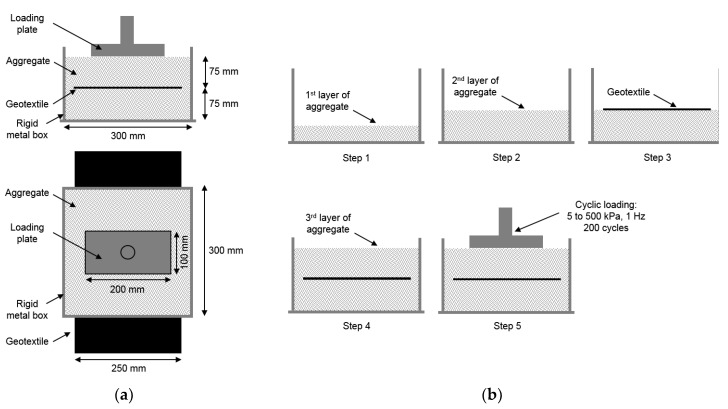
Mechanical damage under repeated loading test: (**a**) equipment, (**b**) damage procedure.

The MD tests were carried out in prototype equipment (in compliance with the requirements of EN ISO 10722 [9]) developed at the Faculty of Engineering of the University of Porto (a full description of the equipment can be found in Lopes and Lopes 2003 [25]). The equipment was formed by a test container (rigid metal box), a loading plate, and a compression machine (Figure 1a). The test container, used to accommodate the aggregates and the test specimens, had internal dimensions of 300 mm × 300 mm. The loading plate (length of 200 mm and width of 100 mm) had the adequate stiffness to transmit the loading forces to the aggregates without deflection. 

The procedure of the MD tests can be divided into five steps (Figure 1b). The layer of aggregate (75 mm high) placed under the geotextile specimen (steps 1 and 2) consisted of two sublayers, each with a height of 37.5 mm. Each sublayer was compacted by a flat plate loaded to a pressure of 200 ± 2 kPa, during 60 s, over the whole area of the test container. Step 3 consisted of the installation of the geotextile specimen (width of 250 mm and length of 500 mm) over the compacted aggregate layer, being then covered (in step 4) by a layer of loose aggregate (75 mm high). The final step consisted of applying a cyclic loading between 5.0 ± 0.5 kPa (minimum) and 500 ± 10 kPa (maximum) with a frequency of 1 Hz during 200 cycles. For each geotextile, 60 specimens were submitted to MD tests: 15 specimens per each of the four aggregates (five for tensile tests, five for puncture tests, and five for water permeability tests).

**Figure 2:**
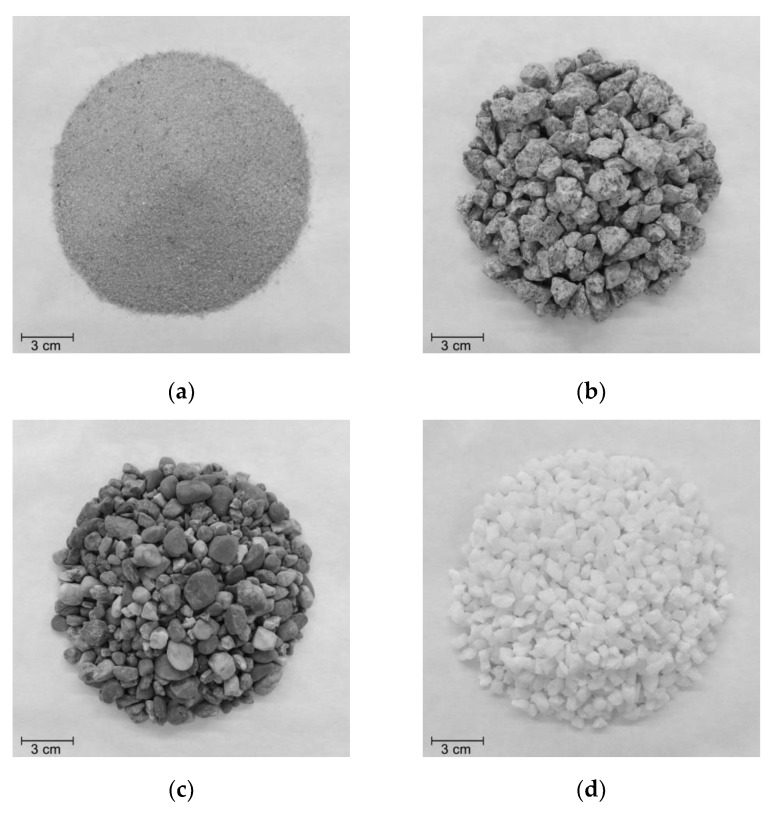
Aggregates used in the MD: (**a**) sand 0/2, (**b**) gravel 6/14, (**c**) river gravel, (**d**) *corundum*.

**Figure 3:**
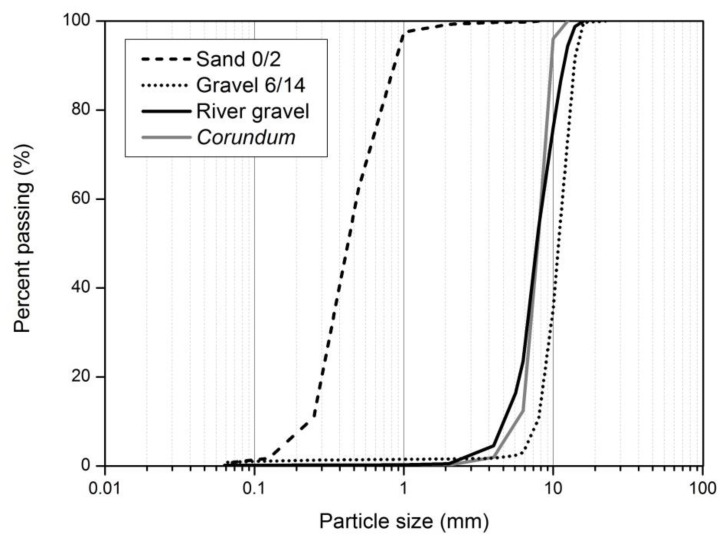
Particle size distributions of aggregates used in the MD tests.

The aggregates used in the MD tests were *corundum* (synthetic aggregate of aluminum oxide considered in the method described in EN ISO 10722 [9]) and three natural aggregates: sand 0/2, gravel 6/14, and river gravel (Figure 2). The particle size distributions of the aggregates (determined according to EN 933-1 [26]) can be found in Figure 3. Table 2 presents some parameters related to the particle size distributions of the aggregates illustrated in Figure 3, where D_10_ represents the effective particle size, D*_x_* represents the particle size corresponding to *x*% passing (*x* = 30, 50, or 60) and D_Max_ represents the maximum particle size.

### 2.3. Evaluation of Damage

Nonwoven geotextiles with masses per unit area within the range of 100 to 500 g·m^−2^ are often used for the separation of geotechnical materials and filtration of fluids. The materials with higher masses per unit area (over ≈ 300 g·m^−2^) may also be able to accomplish the protection function. Therefore, when considering nonwoven geotextiles, it is important to evaluate the effect of mechanical damage under repeated loading on their mechanical and hydraulic properties. Based on this, the damage suffered by the geotextiles in the MD tests was evaluated by monitoring changes on their tensile and puncture behaviors (mechanical characterization) and on their water permeability behavior normal to the plane (hydraulic characterization). In addition, a visual inspection was also performed to identify defects in the geotextiles induced by the MD tests.

**Figure 4:**
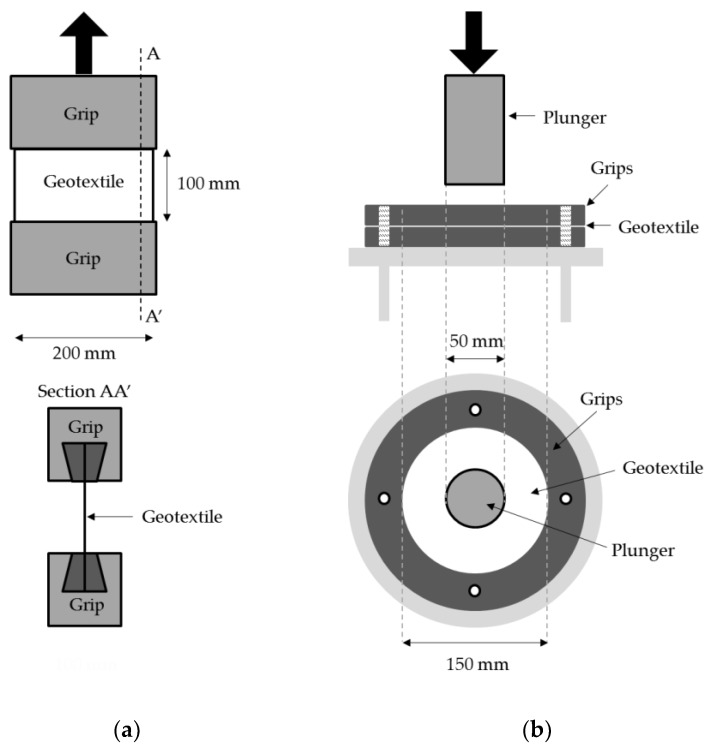
Mechanical characterization tests: (**a**) tensile test, (**b**) puncture test.

The tensile tests and static puncture tests were performed according to EN ISO 10319 [27] and EN ISO 12236 [28], respectively (Figure 4). These tests were conducted in a Lloyd Instruments testing machine (model LR 10K Plus, Bognor Regis, UK) equipped with a load cell of 10 kN (also from Lloyd Instruments). The tensile tests were carried out at 20 mm·min^-1^ using specimens with a length of 100 mm (between grips) and a width of 200 mm (specimens in the machine direction of production). The puncture tests were performed at a speed of 50 mm·min^−1^ using circular specimens (diameter between grips of 150 mm). The plunger pushed through the geotextiles was a stainless-steel cylinder with a diameter of 50 mm (in accordance with EN ISO 12236 [28]). The properties determined in the tensile tests (mean values of 5 specimens) included tensile strength (T, in kN·m^−1^) and elongation at maximum load (E_ML_, in %). Puncture strength (maximum push-through force) (F_P_, in kN) and push-through displacement at maximum force (h_P_, in mm) were the parameters obtained in the puncture tests (also mean values of 5 specimens). The changes that occurred in tensile and puncture strengths are also presented as retained strengths (in %). These retained strengths were obtained by dividing the strengths (tensile or puncture) of the damaged samples (submitted to the MD tests) by the respective strengths of the undamaged samples.

The water permeability behavior normal to the plane of the geotextiles was assessed according to the constant head method described in EN ISO 11058 [29]. In this method, a single unloaded layer of geotextile was subjected to unidirectional flow of water normal to the plane under a range of constant head losses (70, 56, 42, 28, and 14 mm). The water permeability tests comprised the analysis of five specimens with a diameter of 125 mm (83.5 mm exposed to the water flow).

The velocity index for a head loss of 50 mm for a temperature of 20 °C (V_H50_, mm·s^−1^) was the parameter obtained in the water permeability tests. The determination of V_H50_ involved the calculus of the flow velocity value (*v*_20_, in mm·s^−1^) for each head loss and for each of the five specimens. The *v*_20_ was obtained by Equation (1), where V is the water volume (in mm^3^) collected in the time interval t (in seconds), R_T_ is the corrective factor to a water temperature of 20 °C (determined according to EN ISO 11058 [29]) and A is the exposed specimen area (area of 5476 mm^2^).

(1) $$v_{20}=\frac{V\;R_{T}}{A\;t}$$

For each specimen, the different head losses (14 to 70 mm) were plotted against the obtained *v*_20_ and a quadratic curve (passing through the origin of the graph) was adjusted to the data. The V_H50_ corresponds to the flow velocity value at the head loss of 50 mm and was obtained by interpolation using the fitted quadratic regression curves.

The mechanical and hydraulic properties of the geotextiles are presented with 95% confidence intervals calculated according to Montgomery and Runger [30].

## 3. Results and Discussion

### 3.1. Visual Inspection

The MD tests induced different defects on the geotextiles, depending on the aggregates used on those tests and on the physical characteristics of the geotextiles (Table 3). The defects detected in geotextile G120 included cuts in fibers, holes, punctures, abrasion, and stretching (elongation of the specimens). The occurrence and severity of the defects depended on the aggregate used in the MD tests. The MD tests with sand 0/2 caused only some stretching on geotextile G120 (no other types of defects were observed). The other aggregates induced cuts in fibers, holes, abrasion and punctures, being those defects more pronounced after the MD tests with *corundum* (a large number of holes with opening sizes of about 2–3 mm and intense abrasion and punctures). The MD tests with gravel 6/14 and river gravel induced a lower number of holes (compared to *corundum*), but with larger opening sizes (about 4–5 mm). Besides sand 0/2, river gravel was the only aggregate to cause stretching in the nonwoven structure of geotextile G120 (although with lower intensity).

The amount of defects found in geotextiles G300 and G480 after the MD tests was considerably lower than in geotextile G120. The defects observed included cuts in fibers, punctures, and abrasion (no holes or stretching were detected in geotextiles G300 and G480) and their existence and intensity also depended on the aggregate used in the MD tests. Compared to geotextile G120, the intensity of those defects tended to be much less pronounced. The least damaging aggregate for geotextiles G300 and G480 was sand 0/2, being unable to induce any perceptible defects on the materials during the MD tests. Similarly to what happened to geotextile G120, *corundum* tended to be, once again, the most damaging aggregate (higher abrasive effect than gravel 6/14 or river gravel). The increase of mass per unit area (from geotextile G120 to G480) resulted in a significantly better resistance (less amount and intensity of defects) of the geotextiles against mechanical damage. This readily showed that the geotextiles with higher mass per unit area have higher survivability to the MD tests (due to their more robust structure).

### 3.2. Mechanical Properties

The defects reported for the geotextiles in Section 3.1 resulted in some relevant changes in their mechanical behavior (these changes were distinct for the different geotextiles, and also depended on the aggregate used in the MD tests). The tensile and puncture properties of geotextiles G120, G300, and G480, before and after the MD tests, can be found in Table 4.

The tensile and puncture strengths of geotextile G120 suffered considerable reductions after the MD tests, being *corundum* the most damaging aggregate (losses of, respectively, 63.8% and 73.8%). The highest deterioration provoked by *corundum* is in agreement with the amount and severity of the defects found in geotextile G120. Indeed, the significant occurrence of cuts in fibers, holes, punctures, and abrasion weakened the nonwoven structure of geotextile G120, which resulted in a pronounced reduction of its mechanical resistance. As expected, due to the inexistence of pronounced defects other than stretching, the MD tests with sand 0/2 induced only relatively minor reductions on the tensile and puncture strengths of geotextile G120 (losses of 15.7% and 19.1%, respectively). The MD tests with gravel 6/14 and river gravel led to similar reductions in the resistance of geotextile G120 (the effect of these aggregates was in-between the effects of sand 0/2 and *corundum*). This is also in accordance with the defects visually found in geotextile G120. Similarly to what happened for tensile and puncture strengths, the elongation at maximum load and the push-through displacement at maximum force also suffered reductions after the MD tests. The reduction trend observed for these properties tended to be identical to what was observed for tensile and puncture strengths (in terms of hierarchizing the most damaging aggregates).

The tensile and puncture properties of geotextile G300 also suffered relevant reductions after the MD tests. However, and compared to geotextile G120, the deterioration of the mechanical properties was less pronounced. For example, and regarding the MD tests with *corundum*, the losses in tensile and puncture strengths of geotextile G300 (30.2% and 35.0%, respectively) were significantly less relevant than those observed for geotextile G120 (63.8% and 73.8%, respectively). The elongation at maximum load and push-through displacement at maximum force of geotextile G300 also suffered smaller reductions after the MD tests compared to geotextile G120. This greater resistance of geotextile G300 can be ascribed to its higher mass per unit area, which granted considerably better survivability of its nonwoven structure against the damaging actions.

Similarly to what was observed for geotextile G120, the reductions that occurred in the mechanical properties of geotextile G300 were also highly dependent on the aggregates used in the MD tests, being gravel 6/14, river gravel and *corundum* the most damaging aggregates and sand 0/2 the least damaging one. It is worthy to highlight that for geotextile G300 the effects of gravel 6/14, river gravel, and *corundum* were very identical (losses in tensile strength between 28.8% and 30.2% and losses in puncture strength between 30.3% and 35.0%). It is also important to refer that the changes that occurred in the mechanical properties of geotextile G300 are in accordance with the defects detected on its nonwoven structure.

Regarding geotextile G480, some reductions (although less pronounced than those observed for geotextiles G120 and G300) also occurred on its mechanical properties. These reductions tended to be slightly lower than those occurred in geotextile G300, which already presented a relatively good resistance against the MD tests. In this case, the increase of mass per unit area (from 325 to 482 g·m^−2^) resulted only in a small increase of the resistance against the damaging actions. For example, after the MD tests with *corundum*, losses of 30.2% and 29.3% were, respectively, observed on the tensile strength of geotextiles G300 and G480. Compared to tensile strength, the reductions occurred in the puncture strength of geotextile G480 after the MD tests were not much different (slightly higher).

**Figure 5:**
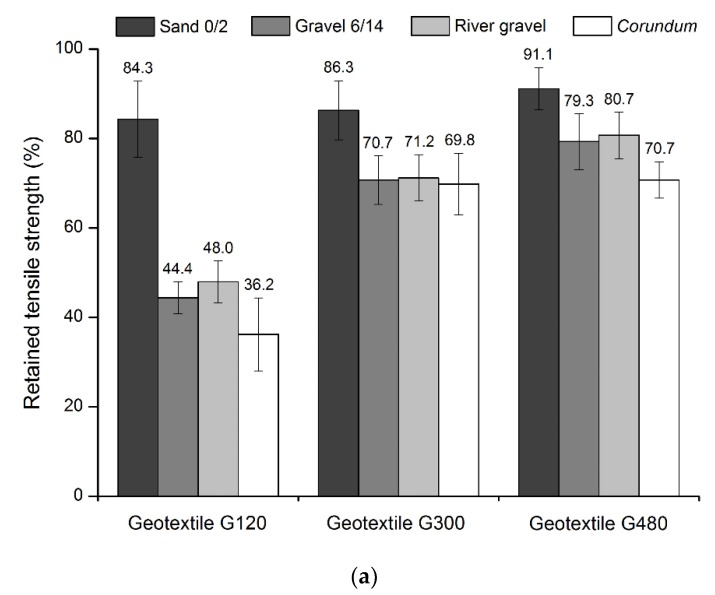

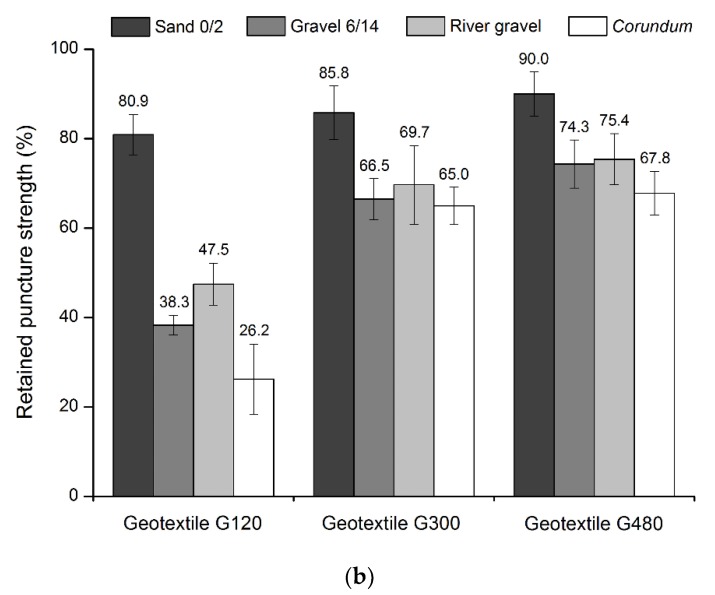
Comparison of the mechanical strengths of geotextiles G120, G300, and G480 after the MD tests: (**a**) retained tensile strength, (**b**) retained puncture strength.

In order to compare the effect of the different aggregates on the mechanical damage suffered by the geotextiles, Figure 5 illustrates the retained tensile and puncture strengths of the materials after the MD tests. It can be easily observed that geotextile G120 was the most affected geotextile (higher deterioration of tensile and puncture strengths) and that *corundum* tended to be the most damaging aggregate. *Corundum* was formed by rough and angular particles, which had a high abrasive effect. This way, it induced severe defects in the nonwoven structures (as can be seen in Table 3), which resulted in serious changes in the mechanical behavior of the geotextiles.

The effects of gravel 6/14 and river gravel were not much different, being gravel 6/14 slightly more damaging to the geotextiles. Despite being formed by larger particles than *corundum* (as can be seen in Table 2 and Figure 3), the constituent particles of gravel 6/14 were less angular, less rough and had a lower abrasive effect. This explains the lower damage (deterioration of the mechanical properties of the geotextiles) induced by gravel 6/14 compared to *corundum*.

River gravel had smoother, rounder, and slightly smaller particles than gravel 6/14 (Figure 2 and Figure 3). However, the river gravel particles are more prone to crack during the MD tests (compared to gravel 6/14), resulting in particles with lower dimension, but significantly more angular and with a higher abrasive effect. This may help to explain the similar results obtained in the MD tests with river gravel and gravel 6/14.

The MD tests with sand 0/2 led to the lowest reductions in the tensile and puncture strengths of the geotextiles (retained strengths above 80%). The only defect detected after the MD tests with sand 0/2 was stretching, and it was only found in geotextile G120. This aggregate had a low capacity to bear the loads imposed by the MD tests and, consequently, the geotextile specimens became concave and were subjected to tensile loads (which caused elongation) while settlements were developed on the lower compacted layer of sand 0/2. Despite the absence of visible defects, the MD tests with sand 0/2 also led to some reductions on the tensile and puncture properties of geotextiles G300 and G480.

### 3.3. Water Permeability Normal to the Plane

**Figure 6:**
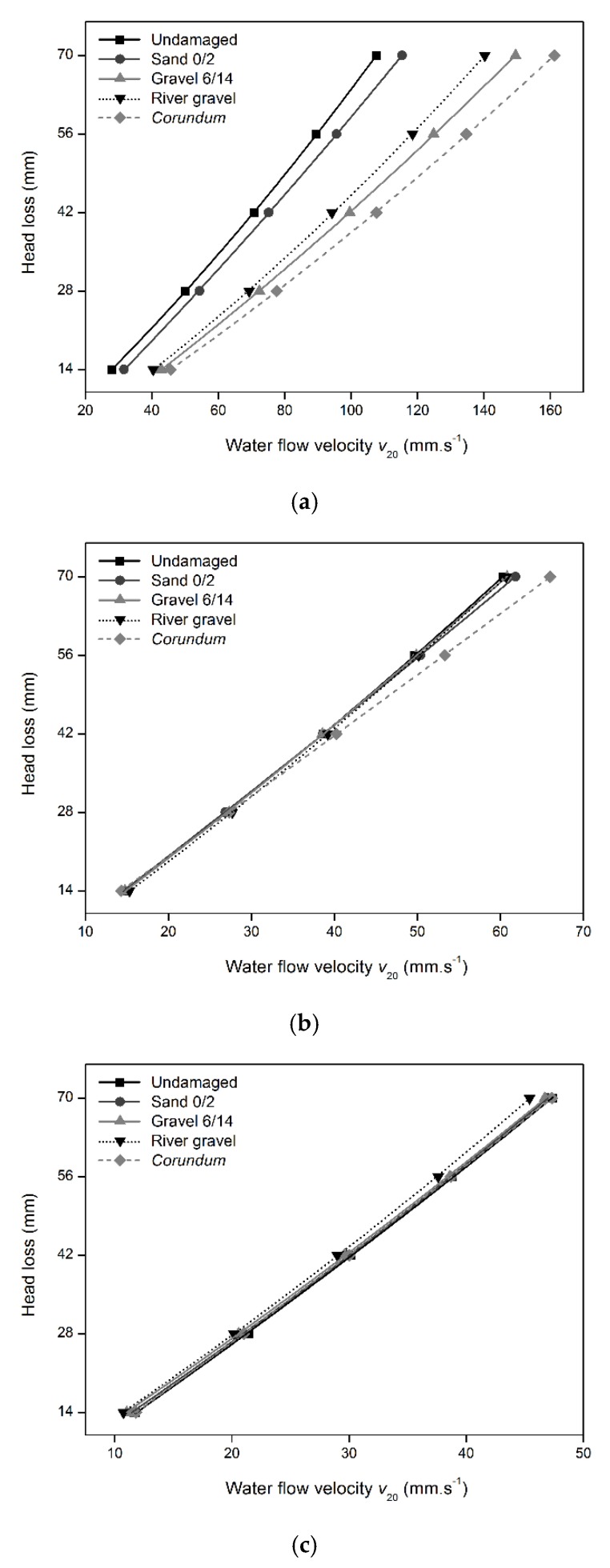
Mean curves “head loss versus *v*_20_” of the geotextiles, before and after the MD tests: (**a**) geotextile G120, (**b**) geotextile G300, (**c**) geotextile G480.

In addition to the changes observed for the mechanical properties, the MD tests also provoked some relevant changes in the water permeability behavior normal to the plane of the geotextiles. These changes can, once again, be explained by the amount of degradation found in the nonwoven structures, which, as previously discussed, depended on the mass per unit area of the geotextiles and on the characteristics of the aggregates used in the MD tests. The values obtained for the V_H50_ of the geotextiles, before and after the MD tests, can be found in Table 5. Figure 6 compares the mean quadratic curves “head loss versus *v*_20_” for geotextiles G120, G300, and G480, before and after the MD tests.

The water permeability normal to the plane (hereinafter, water permeability) of the undamaged samples of the geotextiles was significantly different, being higher for the geotextile with lower mass per unit area. The increase of mass per unit area resulted in a decrease of the water permeability of the geotextiles. Indeed, the water permeability of geotextile G120 was, respectively, about 1.8 and 2.3 times higher than the permeability of geotextiles G300 and G480.

The water permeability of geotextile G120 increased after the MD tests with gravel 6/14, river gravel and *corundum* (increase more pronounced after the MD tests with *corundum*). These increases can be ascribed to the existence of holes in the nonwoven structure (induced by the MD tests), which promoted the flow of water, leading to an increase of the water permeability. Contrarily to the previous aggregates, the MD test with sand 0/2 only led to a small increase of the V_H50_. However, and having into account the 95% confidence intervals, it is not possible to conclude whether this increase reflects the deterioration of geotextile G120 during the MD tests (only stretching was detected, which may have increased the size of the pores of the nonwoven structure, allowing a higher water flow) or if it only represents the typical heterogeneity often found in nonwoven geotextiles.

Contrarily to what happened for geotextile G120, no relevant changes were found on the water permeability behavior of geotextiles G300 and G480 after the MD tests. Indeed, the slight variations found in the V_H50_ are negligible having into account the 95% confidence intervals. These results are consistent with the absence of holes in the geotextiles after the MD tests (therefore, not promoting a higher water flow through the materials). In addition, the presence of other defects (cuts in fibers, punctures, and abrasion) apparently did not have a significant contribution to enhancing the water permeability behavior of the geotextiles.

The defects caused by the MD tests had a different influence on the mechanical and hydraulic properties of the geotextiles. The existence of holes was the main reason for the increase of the water permeability of the geotextiles and may also have contributed to the reductions observed in their tensile and puncture strengths. The occurrence of cuts in fibers, punctures, and abrasion during the MD tests had a notorious impact on the deterioration of the mechanical behavior of the geotextiles. By contrast, their impact on the water permeability behavior of the geotextiles was not clear.

**Figure 7:**
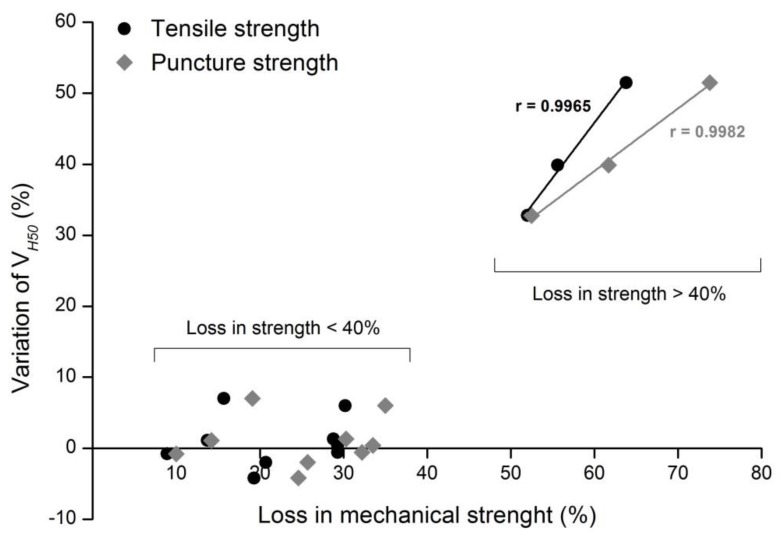
Relation between the loss in mechanical strength and the variation of V_H50_.

Figure 7 presents a relation between mechanical resistance (tensile and puncture strengths) and water permeability (regardless of the geotextiles or aggregate used in the MD tests). The analysis of Figure 7 allows to conclude that the geotextiles whose losses in mechanical strength were lower than 40% had no relevant changes in their water permeability behavior (changes under 5% in most cases). However, when the losses on mechanical strength were higher than 40% (reflecting the existence of considerable degradation in the nonwoven structure), relevant changes also occurred on the V_H50_ of the geotextiles. Despite the relatively low amount of results, it is interesting to note the existence of linear correlations between the increase of the V_H50_ and the losses that occurred in tensile and puncture strengths. The relation shown in Figure 7 confirms that the defects that occurred during the MD tests can affect the mechanical and hydraulic properties of the geotextiles distinctively.

## 4. Conclusions

The MD tests with different aggregates provoked changes in the tensile and puncture strengths and in the water permeability behavior of three nonwoven geotextiles. These changes were a consequence of the damage induced by the MD tests, which depended on the mass per unit area of the geotextiles and on the characteristics of the aggregates used in those tests. The increase of mass per unit area resulted in higher resistance of the geotextiles against the MD tests. Regarding the different aggregates, *corundum* tended to be the most damaging. Therefore, its use in EN ISO 10722 [9] seems to be a conservative approach to evaluate the mechanical damage suffered by geotextiles. The correlation found between the loss in mechanical strength and the water permeability showed that, for resistance changes below 40%, the hydraulic behavior of the nonwoven geotextiles suffered no relevant impact. If not properly accounted, the occurrence of changes in the properties of the geotextiles (many times unavoidable during the installation process) may prevent these materials from correctly performing their functions, which may compromise safety and/or limit the lifetime of the structures in which they are inserted. 

## Figures and Tables

**Table 2:** Parameters related to the particle size distribution of the aggregates.

Aggregate	% < 0.063 mm	D_10_ (mm)	D_30_ (mm)	D_50_ (mm)	D_60_ (mm)	D_Max_ (mm)
Sand 0/2	0.61	0.24	0.34	0.44	0.49	1.0
Gravel 6/14	0.84	7.85	9.56	10.88	11.56	14.0
River gravel	0.19	4.73	6.66	7.80	8.57	12.5
*Corundum*	0.06	5.77	7.05	7.91	8.36	10.0

**Table 3:** Defects observed in the geotextiles after the MD tests.

Geotextile	Aggregate	Types of Defects				
		Cuts in Fibers	Holes	Punctures	Stretching	Abrasion
G120	Sand 0/2	-	-	-	+++	-
	Gravel 6/14	++	++	+++	-	+
	River gravel	++	++	+++	+	+
	*Corundum*	++	+++	+++	-	++
G300	Sand 0/2	-	-	-	-	-
	Gravel 6/14	+	-	++	-	+
	River gravel	+	-	++	-	+
	*Corundum*	+	-	++	-	++
G480	Sand 0/2	-	-	-	-	-
	Gravel 6/14	+	-	+	-	+
	River gravel	+	-	+	-	+
	*Corundum*	+	-	+	-	++

- not detected, + detected (the quantity of + indicates the intensity of the defect (from + to +++)).

**Table 4:** Tensile and puncture properties of the geotextiles before and after the MD tests.

Geotextile	Degradation Test	T (kN·m^−1^)	*E*_ML_ (%)	F_P_ (kN)	h_P_ (mm)
G120	Undamaged	9.30 (±0.46)	39.7 (±5.0)	1.41 (±0.07)	46.0 (±2.4)
	MD with sand 0/2	7.84 (±0.91)	31.3 (±4.7)	1.14 (±0.05)	40.6 (±1.2)
	MD with gravel 6/14	4.13 (±0.36)	23.8 (±2.2)	0.54 (±0.02)	35.8 (±1.7)
	MD with river gravel	4.46 (±0.50)	24.5 (±1.9)	0.67 (±0.07)	36.9 (±1.7)
	MD with *corundum*	3.37 (±0.93)	19.1 (±1.6)	0.37 (±0.14)	32.3 (±0.9)
G300	Undamaged	23.44 (±1.33)	138.4 (±13.8)	4.66 (±0.15)	65.6 (±4.1)
	MD with sand 0/2	20.23 (±1.53)	100.7 (±7.2)	4.00 (±0.32)	55.3 (±2.1)
	MD with gravel 6/14	16.58 (±1.27)	88.8 (±7.9)	3.10 (±0.25)	55.1 (±3.1)
	MD with river gravel	16.68 (±1.17)	81.8 (±7.6)	3.25 (±0.50)	51.9 (±3.5)
	MD with *corundum*	16.36 (±1.79)	81.0 (±7.6)	3.03 (±0.22)	54.4 (±2.4)
G480	Undamaged	34.32 (±1.54)	81.6 (±5.5)	6.70 (±0.31)	55.3 (±0.5)
	MD with sand 0/2	31.28 (±1.44)	78.6 (±4.8)	6.03 (±0.31)	53.2 (±1.2)
	MD with gravel 6/14	27.20 (±2.41)	62.6 (±9.4)	4.98 (±0.38)	48.9 (±1.7)
	MD with river gravel	27.68 (±1.81)	62.2 (±9.6)	5.05 (±0.41)	48.8 (±0.6)
	MD with *corundum*	24.25 (±1.33)	52.6 (±4.7)	4.54 (±0.35)	47.0 (±1.2)

(95% confidence intervals in brackets).

**Table 5:** V_H50_ of the geotextiles before and after the MD tests.

Test	V_H50_ (mm·s^−1^)		
	Geotextile G120	Geotextile G300	Geotextile G480
Undamaged	82.2 (±11.2)	45.2 (±6.8)	35.5 (±2.9)
MD with sand 0/2	88.0 (±12.2)	45.7 (±4.2)	35.2 (±3.8)
MD with gravel 6/14	115.0 (±18.7)	45.4 (±4.7)	34.8 (±5.6)
MD with river gravel	109.2 (±17.6)	45.8 (±7.2)	34.0 (±2.6)
MD with *corundum*	124.5 (±15.8)	47.9 (±3.8)	35.3 (±2.6)

(95% confidence intervals in brackets).
